# Association of BUD13 polymorphisms with metabolic syndrome in Chinese population: a case-control study

**DOI:** 10.1186/s12944-017-0520-8

**Published:** 2017-06-28

**Authors:** Lili Zhang, Yueyue You, Yanhua Wu, Yangyu Zhang, Mohan Wang, Yan Song, Xinyu Liu, Changgui Kou

**Affiliations:** 1 Department of Epidemiology and Biostatistics, School of Public Health, Jilin University, No. 1163 Xinmin Street, Changchun, Jilin province 130021 China; 2 Division of Clinical Epidemiology, First Hospital of Jilin University, Changchun, Jilin 130021 China

**Keywords:** Metabolic syndrome, BUD13, Single nucleotide polymorphism

## Abstract

**Background:**

BUD13 homolog (BUD13), one of submits of the retention and splicing complex, was identified in yeast as a splicing factor that affected nuclear pre-mRNA retention. While more and more studies demonstrated that BUD13 played a potential role in the pathogenesis of metabolic syndrome (MetS). This objective was to reassess whether novel locus of BUD13 were linked to MetS and individual complements in the northeast of China.

**Methods:**

A total of 3850 individuals were recruited in this case-control study, including 1813 MetS cases and 2037 healthy controls. The diagnostic criteria was according to the International Diabetes Federation (IDF). Metabolic complements such as waist circumference (WC), triglyceride, high-density lipoprotein cholesterol (HDL-C), systolic and diastolic blood pressure (SBP and DBP), and fasting glucose were measured. We explored the association between two novel single nucleotide polymorphism (SNPs) of BUD13 (rs7118999 and rs10488698) and MetS and its complements.

**Results:**

Using binary logistic regression analysis we found that there were no significant associations between SNPs and MetS in different heritance models (all *P* > 0.05). However, novel locus of BUD13 were linked to individual complements in MetS cases. Rs7118999 conferred to risk of WC (*P* = 0.016) and the carrier of TT might have higher susceptibility to MetS. While rs10488698 was associated with HDL-C (*P* = 0.001) and the carrier of TT was significantly associated with higher level of HDL-C.

**Conclusions:**

We concluded that novel mutations in BUD13 did not confer risk for MetS in our study population, but these mutations changed the level of metabolic complements.

## Introduction

Metabolic syndrome (MetS) is a cluster of metabolic abnormalities, including raised triglyceride levels, low high-density lipoprotein cholesterol levels, raised blood pressure, and raised glucose levels [1]. Available evidences show that MetS is strongly increasing the risk of developing cardiovascular diseases, type 2 diabetes and all caused mortality [2–4]. Due to escalating prevalence rates and its risk for the development of several chronic diseases, MetS has become the most important health challenge at the global scale [3].

Previous studies indicated that the pathogenesis of MetS might be caused by genetics background, environmental factors and gene-environment interaction [2, 5, 6]. Furthermore, Henneman et al. [7] found that the heritability of MetS based on family study was 10.6%, indicating that gene played essential in the development of MetS. Knowledge of exact genetic factors underlying MetS development might help to explain the etiology of MetS. BUD13 was submits of the retention and splicing complex in yeast [8], while Lin et al. [5] demonstrated that its variant significantly influenced on human development of MetS. Furthermore, Meta-analysis indicated multiple genes linking to MetS, mostly of genes involving in lipids levels, and the heritability of individual components of MetS were range from 21.9–42.9% [7, 9]. Increased studies noted that BUD13 involved in lipid metabolism [5, 10, 11], suggesting BUD13 might played an essential role in the pathogenesis of MetS and its traits through changing lipid levels.

To the best of our knowledge, we selected novel SNPs (rs7118999 and rs10488698) of the BUD13 to evaluate their association with MetS and MetS complements in a sample of the Jilin province, using a case-control study design.

## Materials and methods

### Study population

This study incorporated subjects from Jilin province in the northeast of China, in order to evaluate whether novel locus of BUD13 was linked to MetS and individual complements. The study of community-based consisted of 3850 participants, including 1813 MetS and 2037 non-MetS. MetS was diagnosed according to IDF criteria [12], Which required that subjects with three or more of the following conditions were diagnosed as MetS a) Central obesity with a waist circumference ≥ 80 cm in females and ≥85 cm in males for Chinese subjects b) Triglycerides ≥ 1.7 mmol/L or using drug treatment to elevate triglycerides c) HDL-C < 1.00 mmol/L in males and <1.30 mmol/L for females, or using drug treatment to reduce HDL-C d) SBP ≥ 130 mmHg and DBP ≥ 85 mmHg, or using antihypertensive drug treatment in a patient with a history of hypertension and e) fasting plasma glucose ≥5.6 mmol/L or using anti-diabetic drug therapy.

The study was approved by the ethics committee of the School of Public Health, Jilin University. All subjects signed the approved informed consent.

### Genotyping

The two SNPs (rs7118999 and rs10488698) of BUD13 were selected using the haploview 4.2 software (http://hapmap.ncbi.nlm.nih.gov/), and the minor allele frequency of the above two SNPs was greater than 0.05 in Chinese population.

DNA was isolated from peripheral blood samples using a commercial DNA extraction kit (Hangzhou, China). SNP genotyping was determined by MALDI-TOF-MS (Sequenom, San, Diego, CA, USA) using the Mass ARRAY system, and completed genotyping reactions were divided into a 384-well spectro CHIP. The detection rate of rs7118999 was 93.1% (1687/1813) in MetS cases, and the detection rate of rs10488698 was 99.8% (1810/1813) in MetS cases.

All statistical analyses were conducted using the SPSS program (version 21.0), and the online SNP Stats (http://bioinfo.iconcologia.net/SNPStats) program. Intergroup comparisons of means using the Student’s *t-*test. We conducted the chi-square test to compare the difference from two categorical data. For each SNP, Hardy-Weinberg disequilibrium was tested by χ2 test with 1 degree of freedom. Binary logistic regression analysis was used to evaluate the association of the chosen SNP with MetS by adjusted age, gender, smoking and drinking. There are three inheritance models in this study, including codominant model (TT vs CT vs CC), dominant model (CT + TT vs CC) and recessive model (TT vs CT + CC). Furthermore, we estimated the association of the investigated SNP with individual components of MetS using general linear model (GLM) by adjusted age, gender, smoking and drinking. *P*-value ≤0.05 was considered statistically significant.

## Results

### Characteristics of the subjects

The characteristics of the study population, 1813 MetS cases and 2037 non-MetS subjects, were shown in Table 1. The prevalence of MetS in our cross-sectional survey was 47.1%. The distribution of age and gender were well matched. Moreover, MetS subjects showed significantly increased risk levels for all of the MetS component variables (Waist circumference, triglyceride, systolic blood pressure and diastolic blood pressure, high density lipoprotein, fasting glucose) and the habit of smoking and drinking (all *P* < 0.001).

**Table 1:** Characteristics of study subjects

Characteristic	Case	Control	t/χ^2^	*P*-value
No. of subjects, n	1813	2037		
Age (years)	49.5 ± 9.7	49.5 ± 9.4	−0.062	0.950
Gender				
Male, n (%)	903(49.8)	1024(50.3)	0.082	0.774
Female, n (%)	910(50.2)	1013(49.7)		
Smoking				
Never	1077	1203	21.902	<0.001
Former	199	144		
Current	537	690		
Drinking				
No	1172	1440	16.803	<0.001
Yes	641	597		
MetS components				
WC(cm)	91.4 ± 8.3	75.0 ± 7.0	−66.045	<0.001
Triglyceride (mg/mL)	3.2 ± 2.6	1.1 ± 0.5	−35.939	<0.001
HDL-C (mg/dL)	1.2 ± 0.3	1.6 ± 0.4	42.628	<0.001
SBP (mm Hg)	144.6 ± 19.1	120.2 ± 15.9	−43.146	<0.001
DBP (mm Hg)	87.8 ± 11.0	74.6 ± 9.5	−39.980	<0.001
Fasting glucose (mg/dL)	6.6 ± 2.4	4.8 ± 1.0	−30.243	<0.001

### Associations with MetS risk and quantitative metabolic traits

The distributions of rs7118999 and rs10488698 conformed to Hardy-Weinberg equilibrium among the subjects (*P* = 0.42, 0.73, respectively). The comparisons of genotype distributions of the polymorphisms in the BUD13 between subjects with and without MetS using different model of inheritance were shown in Table 2. We then explored the association of each SNP and MetS using binary logistic regression analysis of risk factors with adjustment for age, gender, smoking and drinking. In the case and control groups, no significant associations between SNPs and MetS were observed in different heritance models.

**Table 2:** BUD13 association with MetS

SNP	Inheritance model	genotype	case	control	Adjusted OR(95%CI)	*P*-value
rs7118999	Codominant	CC	725	825	1.00	
		CT	771	840	1.03(0.90–1.19)	0.656
		TT	191	254	0.84(0.68–1.04)	0.101
	Dominant	CC	725	825	1.00	
		CT/TT	962	1094	0.99(0.86–1.13)	0.845
	Recessive	CC/CT	1496	1665	1.00	
		TT	191	254	0.82(0.67–1.01)	0.058
rs10488698	Codominant	CC	1543	1733	1.00	
		CT	254	288	0.98(0.82–1.18)	0.823
		TT	13	11	1.33(0.59–3.00)	0.491
	Dominant	CC	1543	1733	1.00	
		CT/TT	267	299	0.99(0.83–1.19)	0.931
	Recessive	CC/CT	1797	2021	1.00	
		TT	13	11	1.33(0.59–3.00)	0.486

OR was adjusted for age, gender, smoking and drinking

As shown in Table 3, we also explored to association between novel SNPs and metabolism complements in with MetS subjects. Rs7118999 associated with WC in MetS cases (*P* = 0.016) and the carr﻿ier of﻿ TT was significantly associated with higher WC. However, rs10488698 was associated with HDL-C in MetS cases (*P* = 0.001) and the carrier of TT was significantly associated with higher level of HDL-C. However, our results did not exhibit association between the two SNPs with the rest of MetS components (*P*>0.05).

**Table 3:** Association between BUD13 with complements

characteristics	CC	CT	TT	*t/χ2*	*P-value*
rs7118999	725	771	191		
WC(cm)	91.8 ± 7.9	90.9 ± 8.8	92.3 ± 8.1	4.139	0.016
Triglyceride (mg/mL)	3.2 ± 2.6	3.3 ± 2.6	3.3 ± 3.0	0.337	0.714
HDL-C (mg/dL)	1.16 ± 0.28	1.16 ± 0.30	1.16 ± 0.30	0.114	0.892
SBP (mm Hg)	145.4 ± 19.6	143.6 ± 18.8	144.8 ± 18.8	1.820	0.162
DBP (mm Hg)	88.1 ± 10.9	87.7 ± 11.0	87.4 ± 10.8	0.826	0.438
Fasting glucose (mg/dL)	6.6 ± 2.3	6.6 ± 2.4	6.8 ± 3.3	0.693	0.500
rs10488698	1543	254	13		
WC(cm)	91.4 ± 8.0	91.5 ± 8.3	90.3 ± 5.1	0.132	0.876
Triglyceride (mg/mL)	3.3 ± 2.6	3.2 ± 2.7	2.6 ± 2.5	0.687	0.503
HDL-C (mg/dL)	1.21 ± 0.33	1.22 ± 0.33	1.27 ± 0.30	6.721	0.001
SBP (mm Hg)	144.5 ± 19.1	144.9 ± 19.2	147.2 ± 19.2	0.018	0.982
DBP (mm Hg)	87.7 ± 10.9	88.3 ± 11.2	83.7 ± 12.1	1.221	0.295
Fasting glucose (mg/dL)	6.6 ± 2.4	6.7 ± 2.7	7.2 ± 3.2	0.432	0.649

*P-value* was adjusted for age, gender, smoking and drinking

## Discussion

This study incorporated subjects from community-based cross-sectional study with a sample of Jilin province in the northeast of China. According to the IDF diagnostic criteria [12], the prevalence of MetS was 47.1% in Jilin province in 2012. This prevalence was higher than the prevalence reported in China in 2010 (33.9%) [13]. Here, we demonstrated that novel mutations in BUD13 did not confer risk for MetS among Jilin population, but these mutations changed the level of metabolic complements.

In this literature, we for the first time explored association between novel SNPs in BUD13 and MetS. It has been noted that genetic variants are linking to the development of MetS in different populations. Previous literatures showed that SNPs of rs10790162 [10, 14, 15], rs11216129 [5] and rs623908 [5] contributed to the susceptibility for MetS in Chinese [1, 2], India [3] and Taiwanese population [5]. In this study, the distribution of genotype frequencies of rs7118999 and rs10488698 was no difference between subjects with and without MetS in different model of inheritance (*P* > 0.05).

Furthermore, novel locus of BUD13 were linked to individual complements in MetS cases. The carrier of TT in rs7118999 conferred to risk of MetS by increasing the level of WC. While rs10488698 might a protected factor by increasing the level of HDL-C. Similarly, many studies also investigated that BUD13 variants associated with triglyceride [5, 10, 14, 16, 17], LDL [14], total cholesterol [17] and HDL-C [5], but not discovered rs12286037 and rs28927680 with HDL in Finish [18]. Therefore, the correlation of BUD13 variants with serum lipid levels was not yet clear. Firstly, factors like age, gender, ethnicity, lifestyle and genetic background influenced the association between SNPs with serum lipid levels [19]. Secondly, inter-genetic variant might play an important role in the level of serum lipid [20]. The gene regions of APOA1/C3/A4/A5/BUD13 and BUD13/ZNF were significantly influencing the association with lipid metabolism [10, 16, 21–23].

There are certain limitations to our study. Firstly, our studies subjects were coming from the cross-sectional study, which might limit its ability to detect association between BUD13 and MetS, largely because of bias [24]. Secondly, the etiology of MetS might be caused by multiple factors, such as genetics background, nutritional status and environmental factors. Our study only discussed associations between gene and MetS in the northeast of China, so we could not evaluate the same association in other population. Lastly, the detection rate of SNPs might influence the distribution of MetS and its complements. Therefore, these peculiar characteristics might be contributing factors to the findings of our study.

## Conclusion

We indicated that novel mutations in BUD13 did not confer risk for MetS in our study population, but these mutations changed the level of metabolic complements. The carrier of TT in rs7118999 conferred to risk of MetS by increasing the level of WC. While the carrier of TT in rs10488698 might be protective factor for MetS, who had high level of HDL-C. Considering the complex environment and genetic disease complex mechanism, independent replication studies are needed to provide further insights into the role of the *BUD13*.

## Abbreviations

DBP: diastolic blood pressure
HDL-C: high-density lipoprotein cholesterol
MetS: metabolic syndrome
SBP: systolic blood pressure
SNP: single nucleotide polymorphism
WC: waist circumference
